# Compressive strength after blast of sandwich composite materials

**DOI:** 10.1098/rsta.2013.0212

**Published:** 2014-05-13

**Authors:** H. Arora, M. Kelly, A. Worley, P. Del Linz, A. Fergusson, P. A. Hooper, J. P. Dear

**Affiliations:** 1 Department of Mechanical Engineering, Imperial College London, London SW7 2AZ, UK; 2 FAC Technology, Canterbury Court, 1–3 Brixton Road, London SW9 6DE, UK

**Keywords:** blast, composites, sandwich materials, compression after impact

## Abstract

Composite sandwich materials have yet to be widely adopted in the construction of naval vessels despite their excellent strength-to-weight ratio and low radar return. One barrier to their wider use is our limited understanding of their performance when subjected to air blast. This paper focuses on this problem and specifically the strength remaining after damage caused during an explosion. Carbon-fibre-reinforced polymer (CFRP) composite skins on a styrene–acrylonitrile (SAN) polymer closed-cell foam core are the primary composite system evaluated. Glass-fibre-reinforced polymer (GFRP) composite skins were also included for comparison in a comparable sandwich configuration. Full-scale blast experiments were conducted, where 1.6×1.3 m sized panels were subjected to blast of a Hopkinson–Cranz scaled distance of 3.02 m kg^−1/3^, 100 kg TNT equivalent at a stand-off distance of 14 m. This explosive blast represents a surface blast threat, where the shockwave propagates in air towards the naval vessel. Hopkinson was the first to investigate the characteristics of this explosive air-blast pulse (Hopkinson 1948 *Proc. R. Soc. Lond. A* **89**, 411–413 (doi:10.1098/rspa.1914.0008)). Further analysis is provided on the performance of the CFRP sandwich panel relative to the GFRP sandwich panel when subjected to blast loading through use of high-speed speckle strain mapping. After the blast events, the residual compressive load-bearing capacity is investigated experimentally, using appropriate loading conditions that an in-service vessel may have to sustain. Residual strength testing is well established for post-impact ballistic assessment, but there has been less research performed on the residual strength of sandwich composites after blast.

## 1. Introduction

The recent advances in composite manufacturing have occurred predominantly in the aerospace, marine, automotive and related industries. Formerly, naval vessels were constructed from steel, but composites provide a significant weight reduction and increase in stealth properties while maintaining high strength properties. Glass-fibre-reinforced polymer (GFRP) and carbon-fibre-reinforced polymer (CFRP) composites are quickly finding application in the construction of naval structures as well as new polymer–fibre hybrids. Composites behave in different ways to steels, which have predominantly isotropic properties. This leads to a need for new design protocols to be developed for the safety of naval craft. These materials can be subjected to increasingly demanding and varied conditions during service. In a military context, blast loads represent the most extreme threat to a structure. The research presented in this paper focuses on air-blast loading of sandwich composite panels.

There have been numerous investigations into blast loading of structures using open-air charges and underwater charges since Hopkinson's seminal paper [1]. Several studies have investigated dynamic deformations due to explosive blast loading on plates. Neuberger *et al.*[2,3] and Menkes & Opat [4] highlighted several early studies, which classified the failure modes of structures under impulse loading, from large inelastic deformation to tearing and shear failure at the supports. Neuberger *et al.* also highlighted various studies investigating the scaling effects for comparison of similar blast events using different explosive mass or specimen distance to quantify material response. These studies observed the effect of air blast [2] and underwater charges [3] on clamped circular plates and the validity of scaled testing. Several earlier studies have also investigated the dynamic deformations due to explosive blast loading on plates. Nurick, among others, has conducted extensive studies over the years investigating various plate responses to blast loading [5]. For example, the types of failures described by Menkes & Opat [4] have been investigated further by Nurick *et al.* [6]. In particular, the significant effects of the boundary conditions for the purpose of predicting tearing in steel plates have been highlighted [7]. Cantwell *et al.* [8–10] have continued similar experimental investigations and analysis into composite behaviour under blast conditions. Mouritz [11] has also performed underwater blast testing using 30–50 g explosives on air-backed stitched and unstitched composites in order to determine the reduction of delamination from using stitched composites.

Rather than using explosives to generate shocks, shock tubes have been shown to be a favourable alternative used extensively in shock/blast simulation studies. Tekalur *et al.* [12–14] have experimentally studied the effect of blast loading using shock tubes and controlled explosion tubes loading on E-glass-fibre-based composites and other materials. Arora *et al*. [15] have conducted research, in the past, on the blast resistance of GFRP sandwich panels of varying core thicknesses. The results showed the strong influence of this parameter on the behaviour of the panels, as their stiffness was significantly higher for thicker cores. Wang *et al.* [16–18] performed extensive research on air-blast response of stepwise graded density foam cores and included the addition of a polyurea layer between the skin and core. The effect of these interfaces reduced air-blast damage, and this was also performed with experiments using a shock tube. Wang & Shukla [19] performed further shock tube testing on composite sandwich panels with in-plane compressive loading present during the shockwave loading, in an attempt to replicate the in-plane loading applied to many sandwich composite vessels in service, such as the hull of a ship.

The research presented in this paper adds to previous research, focusing on full-scale air-blast experimentation conducted on GFRP and CFRP sandwich composite panels. These panels were of similar mass per unit area and overall thickness. This provided for a direct comparison between these two forms of construction. The aim was to evaluate these materials experimentally in terms of blast response and damage sustained, and to assess their residual load-bearing capacity, a feature often overlooked.

The residual edgewise compressive strength of the air-blasted panels was evaluated. Arora *et al*. [15,20] performed air blasts of 38.4 kg TNT equivalent against large-scale GFRP sandwich panels as well as 100 kg TNT equivalent blast tests against GFRP and CFRP sandwich panels [21]. Here, strength was evaluated relative to location on the sandwich panel during the blast, i.e. where on a marine vessel or section of a marine vessel is most susceptible to compressive failure in service post-blast. The nature of damage sustained and location of damage were recorded prior to compression, and analysis of these experiments and related numerical studies is the main focus. Strength after impact testing is fairly commonplace in composites research in general, particularly in the area of ballistic impacts. However, there is relatively little research on post-blast damage due to blast loading, as blasts are often considered catastrophic. This research, on the other hand, considers the capability of a naval vessel subjected to blast continuing in limited service, maintaining sufficient residual strength and integrity. When a composite sandwich panel is subjected to blast loading, complex damage conditions can be generated throughout the panel, which are difficult to quantify in order to make an estimate of residual strength of the material for further use. This research looks at crack distributions in sections of the panel, and relates these to the residual strength. Some early research was performed on this by Caprino & Teti [22], where glass-fibre skin–polyvinyl chloride (PVC) core sandwich panels were subjected to impact on a drop tower, and their residual strength determined with tensile testing. Three densities of core were tested to determine the effect of core strength on residual strength, and it was found that the effect was minimal. It was further concluded that the residual strength of the sandwich panels was actually dominated by fibre failures during impact, as opposed to delamination, as was originally suggested.

Bull & Edgren [23] performed residual strength testing on carbon-fibre skin–PVC core sandwich panels, which had been subjected to impact loading on a drop tower. Two impactor shapes were used, a spherical indentor and a pyramid-shaped indentor, in order to create different types of damage in the specimen. Two drop heights were tested, one that would cause minimal damage, and one that would cause severe damage. These test results were then compared with residual strength models, and good correlation was determined, within 6% for the spherical indentor. This model assumes an equivalent circular hole in the panel with cracks emanating from its boundary, with the initial size of the hole determined by C-scans of the initial impact damage. This model was then further developed by Zenkert and co-workers [24], who assumed closing cracks developing from a notch and incorporated cohesive laws to predict crack growth. This showed improved correlation with test results.

The edgewise compression tests performed by Zenkert and co-workers [24] caused panel failure in the facesheet containing the damage from the impactor, as is expected. The actual mode of failure of the panels was rapid growth of the dent left by the impactor into the panel. By using digital image correlation (DIC), these tests were used to verify finite-element analysis (FEA) models, in which damage was already present in the form of a region of already crushed core material directly below the dent in the front facesheet. With the FEA model set up in this way, strong agreement was found with experimental results for panel response in edgewise compression and the onset of dent growth [24]. This approach is similar to that used in this research, in which damage is generated in the FEA geometry from the start, in order to determine the residual strength with this damage in place. Lacy & Hwang [25] also produced FEA models of facesheet and core damage due to impact. In this model, various radii of reduced tensile modulus were incorporated into the facesheet around the point of indentation to account for the reduction in stiffness caused by damage. These properties and the severity of indentation were estimated from non-destructive inspection, such as C-scans. Similarly, a separate section was introduced directly beneath the indented facesheet to account for the reduction in stiffness of the honeycomb core. In this study, it was found that the residual strengths predicted using FEA were significantly less than those found experimentally, but that failure modes were consistent.

Compression after impact testing has also been carried out on carbon-fibre facesheets on an aluminium honeycomb core by Davies *et al*. [26] and, while honeycomb cores differ greatly from the polymeric foam cores studied here, there is some merit in comparing residual strength predictions and test methods. The impact tests performed on these panels used a 20 mm flat indentor on a drop tower and two impact energies. Two types of panel lay-ups were tested to cause top-face and local core damage in half of the panels and debonding of the back face and the core in the others. These impacted sandwich panels showed a 35% reduction in strength for a thin core with back-face delamination, and a 68% reduction with a thick core. These figures are 31% and 18%, respectively, for front-face damage and core crushing.

**Figure 1.**
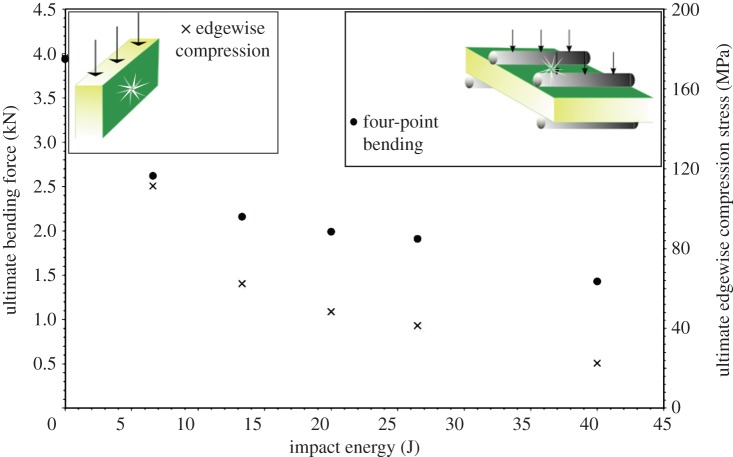
Impact energy to cause inherent damage versus stress to cause failure in edgewise compression, and force to cause failure in four-point bending. Adapted from reference [28]. (Online version in colour.)

Xie *et al*. [27] also performed tests on carbon-fibre facesheets with an aluminium honeycomb core, this time looking only at barely visible damage. A drop tower was used to create mild indentation with ply delamination and core crushing, and then FEA models were created with damage present, to be validated using edgewise compression testing. The delamination of the skin and core was already present in the model, and this was achieved using point-to-surface contact elements. The FEA incorporated routines to simulate in more detail core crushing and the nonlinear effect of the core stiffness during displacement. Very good correlation was found with experimental results and modes of failure with core crushing present, whereas there was high deviation at higher strains without core crushing present. Shipsha *et al*. [28] investigated the effect of impact on a panel by a spherical indentor, and on a beam by a cylindrical impactor. It was found that, in both cases, a cavity formed underneath the skin in the core where the two had completely separated, and core crushing occurred around this zone. The materials used were polymethacrylimide foam cores and glass-fibre skins. In these tests, drop tower energies were used that would cause negligible damage in the front facesheet. This was found to produce a reduction in stiffness of less than 5%. Post-impact testing of these sandwich panels was in the form of edgewise compression and four-point bending. In the bending tests, it was found that failure took place suddenly at the periphery of the local core damage, with no steady crack propagation beforehand. The variation of force to cause failure in the four-point bending tests, and stress to failure in edgewise compression tests, versus the impact energy used to create initial damage are shown in figure 1.

Various approaches have been addressed to develop numerical models for the prediction of failure stress, a topic that is covered in this paper. However, an inherent failure criterion that is missing from the literature is the presence of shear cracks in the core, which cause a discontinuity between the two facesheets, which can be detrimental to the residual strength and stiffness of the sandwich panel. Shear cracking and resultant skin and core debonding are the primary damage mechanisms in blast loading of large-scale sandwich composites. This paper therefore addresses the effect of crack distribution and density after the blast event as well as debond severity on residual properties. Criteria for damage assessment of service life are investigated, with the view of damage mapping models being generated to enable the assessment of continued service applied to naval vessels subjected to blast.

## 2. Materials

The composite materials used in this study were all purchased from S. P. Gurit, manufactured by P. E. Composites. GFRP- and CFRP-skinned sandwich panels were produced, each constructed with a 25 mm thick closed-cell M130 Corecell styrene–acrylonitrile (SAN) foam core. Four composite sandwich panels, 1.6×1.3 m in area, of equivalent mass (26 kg) were evaluated: two with GFRP skins, denoted GL1 and GL2; and two with CFRP skins, denoted CA1 and CA2.

The glass-fibre-skinned panels, GL1 and GL2, were constructed using two plies of 0°/90°/±45° E-glass quadriaxial skins (QE1200) on a 25 mm SAN foam core (M130). The CFRP equivalent panels, CA1 and CA2, consisted of skins with two repeat layers of two plies of 0°/90° carbon (RC245T) and two plies of ±45° carbon (RC380T) on a 25 mm SAN foam core (material code M130). Ampreg 22 was the epoxy resin system used for each test sample. Material properties for all constituent materials used during this study are summarized in table 1.

**Table 1.** Material properties of the GFRP and CFRP sandwich panel constituent elements [29].

material property	QE1200	RC245T	RC380T	M130
density (kg m^−3^)	1750	1393	1393	140
tensile modulus (GPa)	17	50	11	0.18
compressive modulus (GPa)	—	—	—	0.17
tensile strength (MPa)	260	471	—	2.85
compressive strength (MPa)	200	316	—	2.31
shear modulus (MPa)	6500	3150	23 600	59
tensile failure strain (%)	1.5	0.9	—	1.6

## 3. Blast loading of sandwich materials

The research presented in this paper builds on previous studies focusing on full-scale air-blast experimentation conducted on GFRP [15,20] and CFRP [21] sandwich composite panels. This previous research provided experimental data for full-scale explosive testing on composite materials. The nature and quality of scaling from small-scale to large-scale blast response is still in development within the research community. Therefore, large-scale data and novel approaches applied to data acquisition are an important contribution to this field of research, in particular for industry. The experiments presented here were performed at RAF Spadeadam, Cumbria, UK. The Hopkinson–Cranz scaled distance [30,31], *Z*, of 3.02 m kg^−1/3^, 100 kg TNT equivalent at a stand-off distance of 14 m, was used for each of the four targets presented here. The aim of these air-blast experiments was to capture full-field displacement–time histories of the sandwich composite panels and understand how damage develops during a given blast event. High-speed three-dimensional DIC was used to capture full-field displacement plots of the rear surface of the targets. These experiments provide full-scale data to validate analytical and numerical models of such structures.

### (a) Experimental

**Figure 2.**
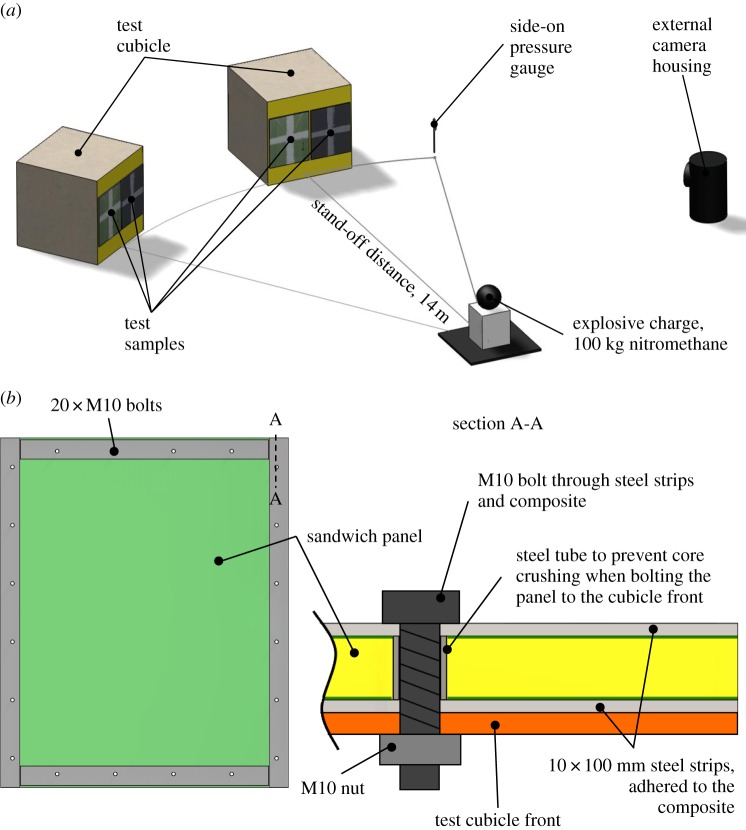
(*a*) Schematic of the experimental layout on the test pad at RAF Spadeadam, Cumbria, UK, and (*b*) the clamping arrangement of the sandwich panels onto the test cubicle. (Online version in colour.)

Damage was induced in the sandwich panels by use of an explosive charge, simulating a moderate blast load in close proximity to a structure in air. Charge size and stand-off distance were chosen, based on a finite-element simulation presented in reference [20], to initiate failure in the GFRP panel and hence allow a comparison of the CFRP panel's performance. A 100 kg charge of nitromethane was assembled 14 m from the front of the test cubicle. The panels were mounted and the charge set up on the mid-plane of the cubicle, with the front face perpendicular to the direction of the charge. A schematic of the full test layout is given in figure 2*a*. In order to create quasi-built-in boundary conditions, the panels were bolted to the test cubicle using 20 M10 bolts around the perimeter. Then, 5 mm thick steel strips were adhered to both sides of the composite panels as shown in figure 2*b*. This clamping approximates the loading as a ‘built-in’ boundary condition. Full modelling should also consider the response of the cubicle front.

Two pairs of Photron SA3 high-speed video cameras were positioned inside the cubicle, each behind one of the two panels tested side-by-side to record back-face deformation in three dimensions. The speckle pattern applied to the back face of each panel was sized according to the 1 MP resolution of the cameras at a frame rate of 2000 fps. The pattern was applied in matte acrylic paint, with an even distribution over the whole panel, but randomly distributed within the frames of the facets used for measuring displacement in the DIC software. The positions of the cameras were accurately recorded by imaging a calibration pattern before the test, once the cameras were in their final positions. LaVision provided the high-speed cameras for this study, and the DIC analysis in this section was performed using the LaVision software [32].

An additional high-speed camera was positioned outside of the cubicle to observe the effects of the blast on the front face of the panel and moreover to allow the user to confirm that the test conditions remained as desired throughout. An arbitrary cross shape was painted onto the front of the panels to aid in the visualization of the deflection during blast, but not intended as a quantitative measurement of the front face. The displacements were taken from the rear face using DIC. Overpressure due to the blast was measured with a pressure gauge positioned next to the cubicle at the same stand-off distance (figure 2*a*).

The samples were tested side-by-side to compare directly the response of the GFRP sandwich panel with the CFRP sandwich panel. For one pair of test samples (GL1 and CA1), central point displacement, full-field contour DIC data and pressure data are provided for the duration of the event. Two pairs of samples tested at the same scaled distance provide good statistical comparison for observing the effect of skin configuration on blast mitigation and damage incurred. It is to be noted that the second pair of composite sandwich panels, GL2 and CA2, were positioned at the same stand-off from the charge, but were not instrumented for DIC analysis. They were simply included to provide more test samples of blasted panels for post-blast evaluation.

### (b) Results and analysis

**Figure 3.**
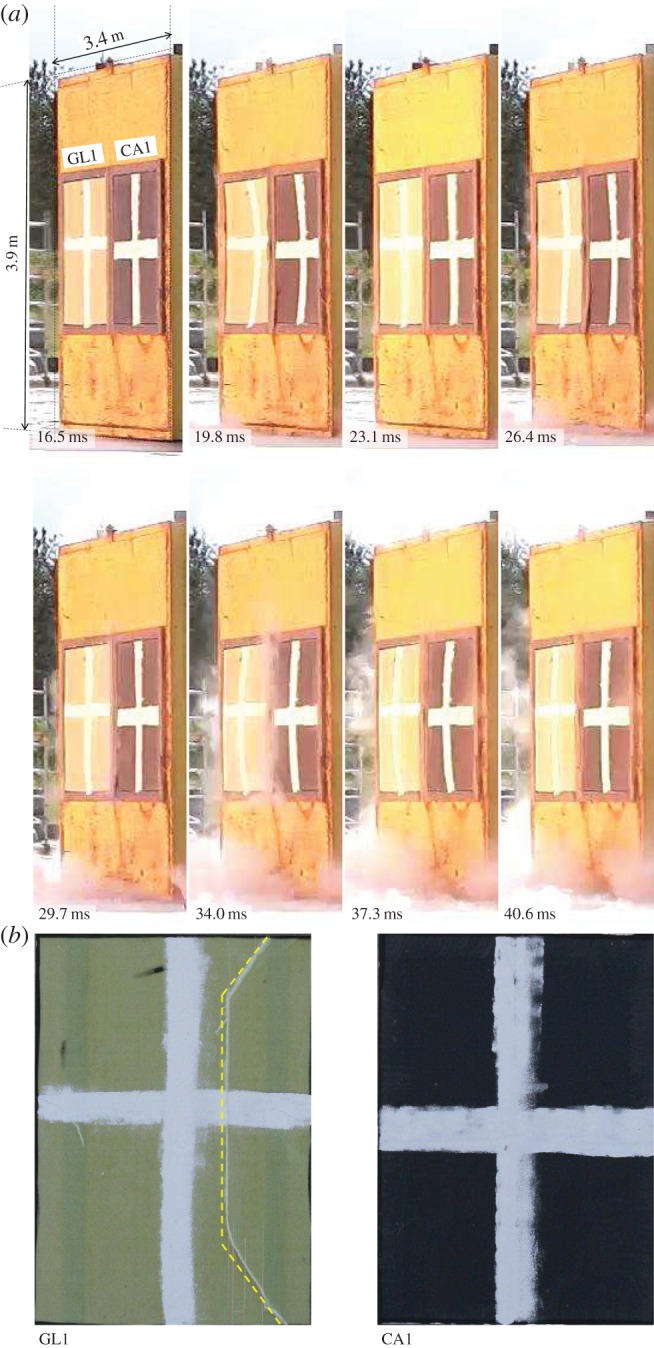
(*a*) Images taken at regular intervals with the shockwave impinging on test samples GL1 and CA1 after 16.5 ms. (*b*) Final visible front-face damage. Time stamps are relative to detonation time at 0.0 ms. (online version in colour.)

**Figure 4.**
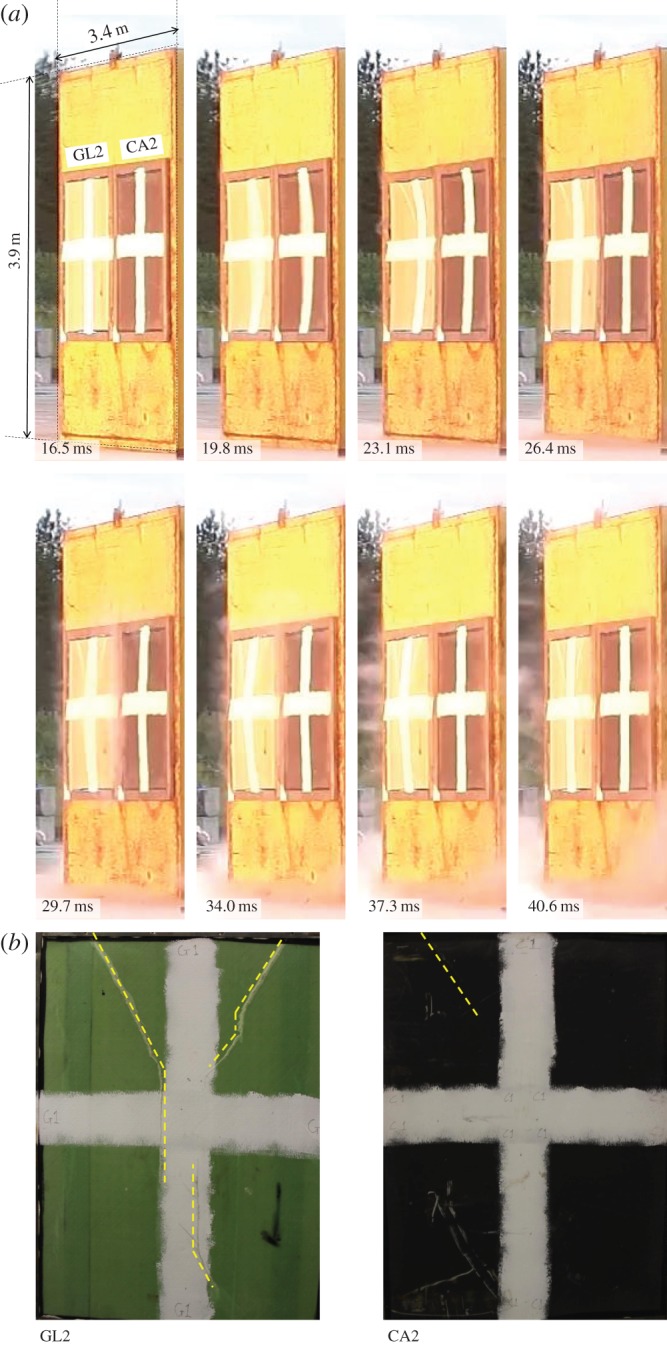
(*a*) Images taken at regular intervals with the shockwave impinging on test samples GL2 and CA2 after 16.5 ms. (*b*) Final visible front-face damage of targets 1.6×1.3 m area. (Online version in colour.)

Figures 3 and 4 show sample front-view images taken from the high-speed video cameras positioned on the test pad during the 100 kg nitromethane charge explosion. The shockwave is seen to arrive at the target after 16.5 ms post-detonation. This blast experiment was designed to take the panels beyond their elastic limit and inflict a significant proportion of damage on each target. The test samples have an arbitrary cross painted on their front face for ease of tracking the front face deformation.

Figure 3 shows the effect of the blast on test samples GL1 and CA1. Front-face damage is seen to initiate in the top right corner of GL1 at 19.8 ms and propagate down the right-hand edge. The final front-face damage is shown at the bottom of figure 3, highlighting the relative visible damage sustained by each target. There was no visible skin damage in CA1.

For the second pair of samples subjected to blast, figure 4 shows the external view of samples GL2 and CA2 for the duration of the loading cycle. Front-face damage is seen to initiate in the top left and right corners of GL2 between 19.8 and 23.1 ms. These cracks propagate down the sides of GL2. This time, in the CFRP panel, there is a small amount of skin failure visible, highlighted in the final front-face damage image at the bottom of figure 4.

**Figure 5.**
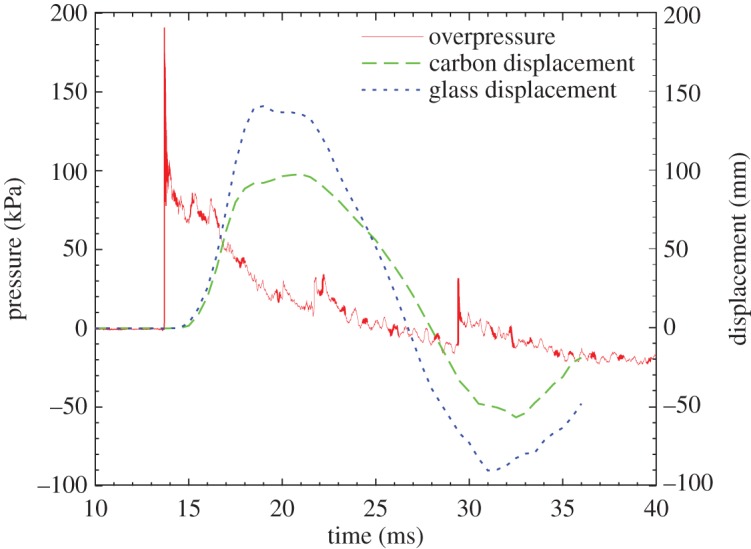
Graph showing the out-of-plane displacement of both CA1 and GL1 alongside the synchronized overpressure measurement. (Online version in colour.)

At the 14 m stand-off from the 100 kg charge, an impulse of 420 kPa ms was recorded, and the initial positive pressure wave lasted for 12.0 ms. This is shown alongside the transient central out-of-plane displacement plots for CA1 and GL1 in figure 5.

**Figure 6.**
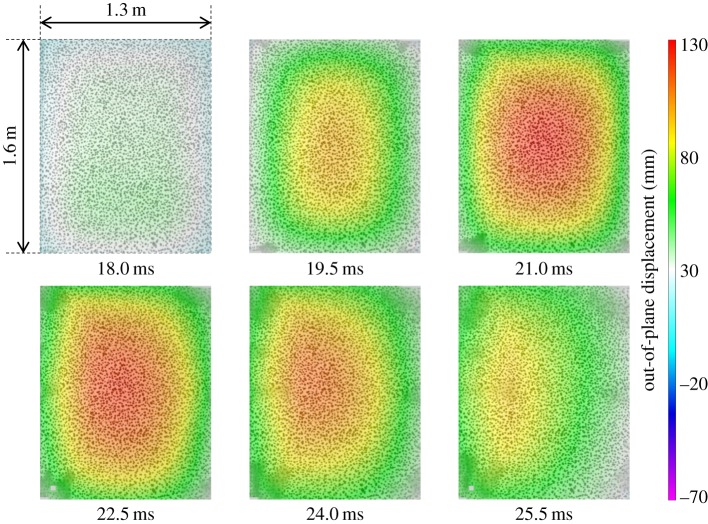
DIC analysis of blast on GL1 featuring contour plots of out-of-plane displacement. (Online version in colour.)

The glass-fibre-skinned panel, GL1, deformed symmetrically until the point of maximum *z*-displacement. By this point, front-face and core damage had already occurred and affected the return response of the panel, causing a non-uniform rebound response at 20 ms about the peak out-of-plane displacement (see graphic in figure 5). This asymmetry caused by the damaged core is highlighted in the out-of-plane displacement contour plots in figure 6.

**Figure 7.**
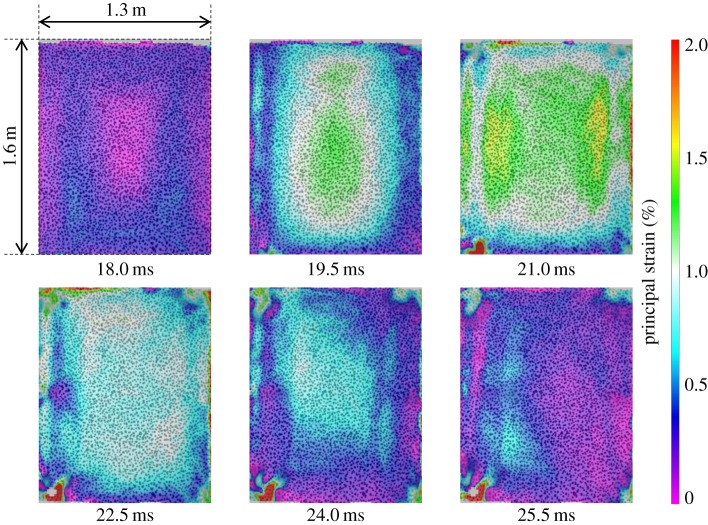
DIC analysis of blast on GL1 featuring contour plots of maximum principal strain. (Online version in colour.)

**Figure 8.**
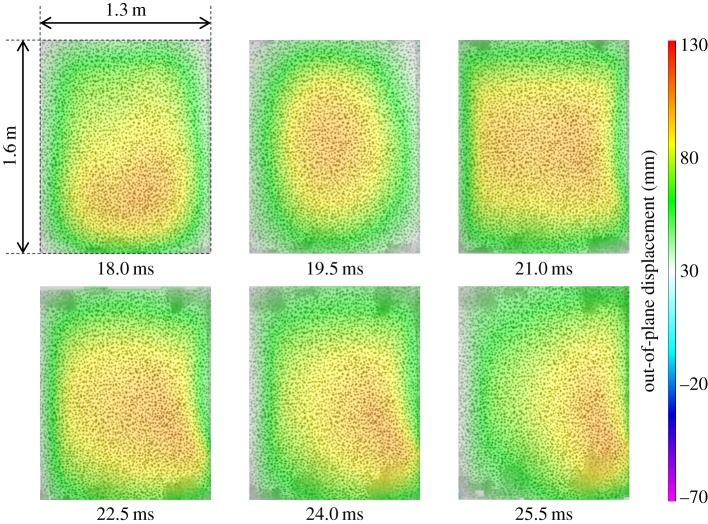
DIC analysis of blast on CA1 featuring contour plots of out-of-plane displacement. (Online version in colour.)

**Figure 9.**
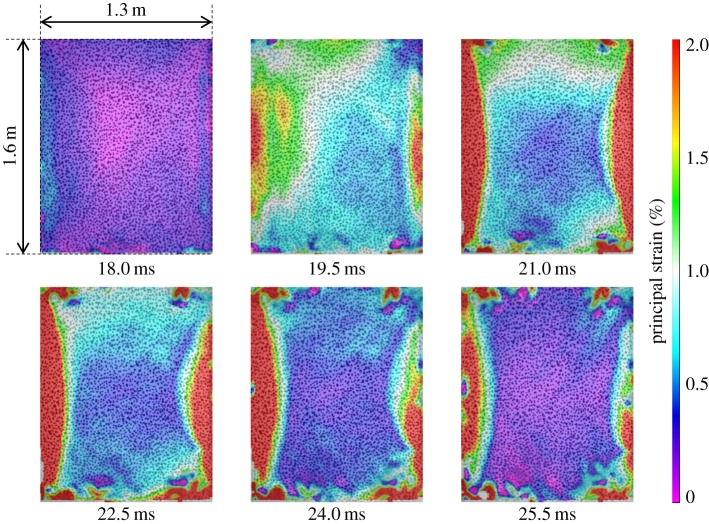
DIC analysis of blast on CA1 featuring contour plots of maximum principal strain. (Online version in colour.)

The maximum *z*-displacement, 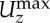, was 140 mm and the maximum principal strain, 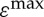, peaked at 1.6% towards the right- and left-hand edges of the panel (figure 7). The carbon-fibre-skinned panel, CA1, deflected to a 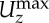 of only 107 mm, owing to a higher stiffness than that of the glass-fibre-skinned panel. Core shear failure was still observed. There was no skin damage visible in CA1, although a fine crack on one of the panel edges of CA2 was observed to occur, as shown previously in figure 4. The damage to the panel also led to a reduced stiffness, which caused an increase in the time for the return response. This was seen in the carbon-fibre panel as an elongated return from maximum displacement in figure 5 and the relatively flat-looking contour plot in figure 8. It took 1 ms longer for the carbon–fibre panel to return to its original position than the glass-fibre panel owing to the difference in core crack distribution observed in the carbon-fibre-skinned panels compared with the glass-fibre-skinned panels and other effects. CA1 was seen to have strain concentrating around the edges of the panel in figure 9, with the central region relatively unstrained, complementing the displacement observations in figure 8. Similarly, the edges of the GL1 were relatively highly strained between 18.0 and 21.0 ms; however, this strain progressed to concentrate around a region that suffered complete failure along an edge (figure 7). This coincided with the time at which a front-face failure was observed in figure 3.

**Figure 10.**
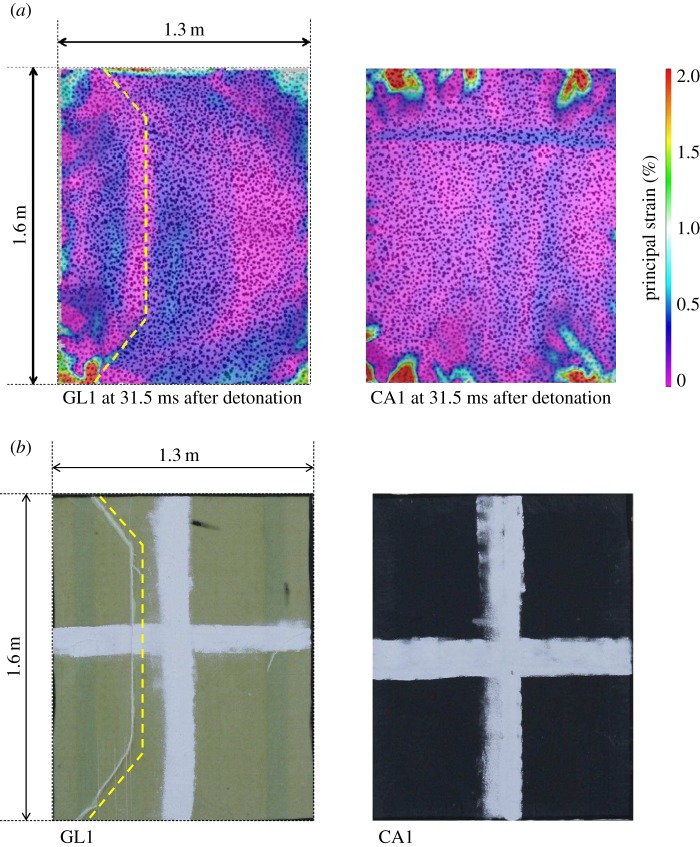
DIC analysis of both GL1 and CA1 showing (*a*) the maximum principal strains and (*b*) the mirrored images of the damaged front faces of each target. (Online version in colour.)

Clear indications of damage induced are shown in the DIC images. Sample snapshots of the maximum principal strain are given in figure 10*a*. For GL1, there is a region of material in compression of approximately −1% when GL1 is at its peak rebound displacement (out towards the origin of the charge). This region builds in strain owing to the lack of support provided by the core. This lack of support due to core cracking can also be observed in CA1, along the top of the panel, but, in this case, front facesheet damage is not observed owing to the higher strength of the carbon fibre. A core shear failure propagated through the panel, resulting in a front-skin failure as well shown in figure 10*b*. The front-face image of the damaged sample in figure 10*b* has been mirrored for ease of comparison with the rear-face DIC result in figure 10*a*. In both carbon panels, there was no visible skin damage apart from a few hairline cracks on the front-most fibres. The higher-stiffness fibres resisted the blast better in terms of a lower out-of-plane deflection. However, by deflecting less, more energy was retained in the sandwich panel and, rather than being dissipated in momentum, it was dissipated in internal reflections within the sandwich material. This led to a greater coverage of core shear failure within the carbon-fibre-skinned panels than the glass-fibre-skinned ones. Evidence in figure 10*a* shows a typical flicker of subsurface damage in CA1. During the deformation and rebound phases, the core cracks are individually highlighted with bands of strain concentration, i.e. the dark (blue online) streaks in the plot of 0.5% strain across the relatively unstrained surface (light (purple online)). Areas where wider bands of strain relief are observed also suffered propagation of the shear cracks into interfacial failures as well, again showing that the strain contour plots available through DIC analysis can be used as an indication of subsurface damage in the sandwich composites (to be discussed further in §3*c*).

### (c) Damage analysis of sandwich panels

**Figure 11.**
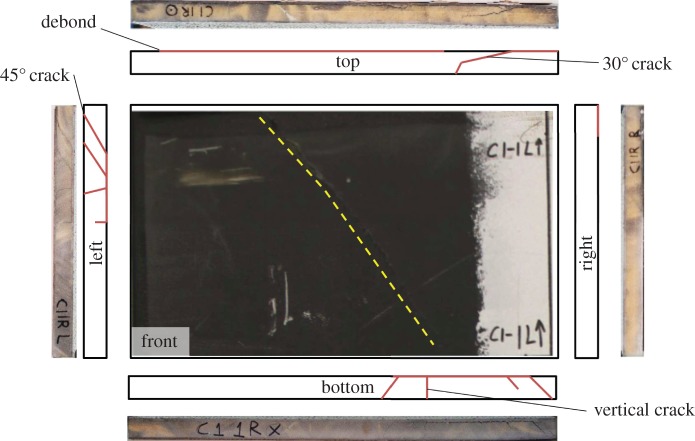
An example of the documentation process for visual inspection post-blast on CA1 subpanel 1R (650×400 mm in area), showing images and schematics of damage noted. Subpanel 1R is top right when viewed from the rear. (Online version in colour.)

**Figure 12.**
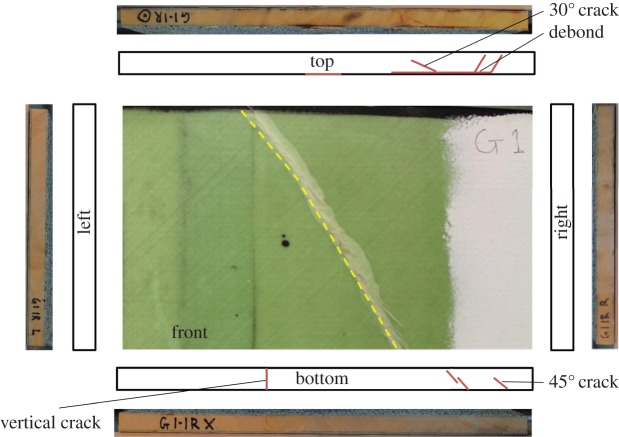
An example of the documentation process for visual inspection post-blast on GL1 subpanel 1R (650×400 mm in area), showing images and schematics of damage noted. Subpanel 1R is top right when viewed from the rear. (Online version in colour.)

After the blast experiments, the samples were recovered and sectioned into eight equally sized subpanels, 635×400 mm in area. The global panels of CA1, CA2, GL1 and GL2 were cut down their vertical into halves and then cut into a further four sections down each side. Each subpanel was then visually inspected for damage. An example of a 1R subpanel (top right when viewed from rear) for CFRP is presented in figure 11 and a 1R subpanel for GFRP is presented in figure 12. Core cracks are identified and recorded in terms of orientation. Debonded surfaces are also recorded in terms of percentage propagation along a length. Sample records are shown for CA1 and GL1 for subpanel location 1R, presented in table 2. Such visual inspection records were collected for all subpanels, to be used later in the discussion to analyse how post-blast damage influences residual strength. The estimates are based on debonds as a percentage of the length of the edge, from edge inspections. This can be further verified with the use of three-dimensional CT scanning to determine the extent of subsurface damage.

**Table 2.** Index of damage observed on the subpanel samples shown in figures 11 and 12.

sample		top edge	bottom edge	right edge	left edge
CA1-1R	front	80% debond	30% debond	15% debond	25% debond
	core	1×45°, 1×30°	3×45°, 1×90°	—	2×45°, 2×90°
	back	—	—	—	—
total no. of cracks		10			
total area of debond		37%			
GL1-1R	front	20% debond	—	—	—
	core	1×45°	2×45°, 2×60°	—	—
	back	—	15% debond	—	—
total no. of cracks		5			
total area of debond		6%

**Figure 13.**
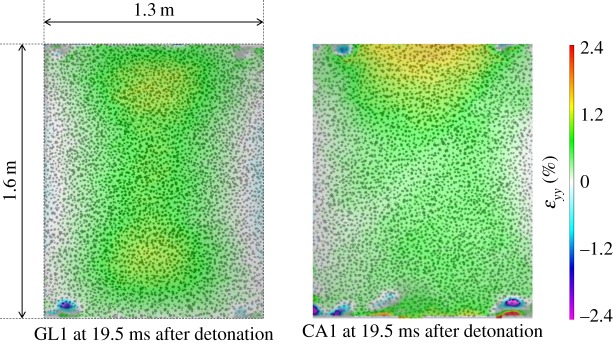
DIC analysis showing strain in the *y*-direction in GL1 and CA1 recorded during the blast taken at 19.5 ms after detonation. (Online version in colour.)

Signs of where the most damaged areas of each global panel occur were available in the DIC analysis. Figure 13 shows the strain in the *y*-direction in the image, denoted *ε*_*yy*_, for GL1 and CA1. It is shown that the propensity for horizontal cracking along the shorter length (*x*-direction) is fairly uniform in GL1, whereas in CA1, *ε*_*yy*_ is much higher towards the top edge of the target. This goes some way to highlight why visual inspections show that the GFRP panels were more uniformly damaged throughout the target compared with the CFRP sandwich panels. Evidence presented in the previous section highlighted the fact that damage initiated at points of high constraint, i.e. the corners of the support structure, and these visual inspections show the extent of through-thickness damage.

## 4. Edgewise compression of sandwich panels

While the blast experiment allows for an assessment of hazard to occupants from the blast-wave energy, it does not account for the changes in load-bearing capacity of the material once damaged. In order to understand the effects of the blast on the structural integrity of the panels, it was decided to test the panels under a compressive load that the panels might be expected to support within a larger structure. It is important to note here that blast tests were not conducted with pre-load applied to the panels. The severity of the damage to the panels and the dynamic response of the panel to the pre-load under these conditions may vary from the idealized situation presented here. Such relevant modifications to the blast experimental set-up are of interest for future investigations. The tests conducted here demonstrate the ability of the panels to support applied load after blast damage has already occurred.

### (a) Experimental

Once recovered from the blast experiment, the carbon- and glass-fibre-skinned panels were each sectioned into eight equally sized samples. The original panels were split down the middle vertically, and then into four smaller panels horizontally. Damage was observed along the edges of each panel, and recorded in a series of images. Examples of such images are shown in figures 11 and 12. These images were used to estimate the degree of damage within each panel to correlate such measures of damage, as presented previously in table 2, with residual strength.

Panels were restrained in 10 mm deep channels aligned between compression plates on an Instron 8806 2.5 MN dynamic hydraulic testing machine. The channels constrained the panels against out-of-plane displacement, but were shallow enough (5 mm edge height restraint) to allow rotation at either end. Low-stiffness elastomeric sheet was inserted at either end of the panel to help ensure even loading along the whole panel length.

**Figure 14.**
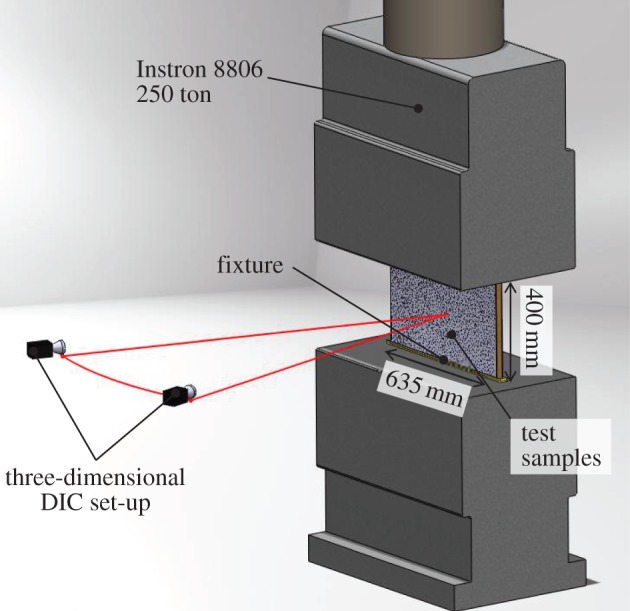
Schematic of the experimental test set-up for edgewise compression of sandwich materials. (Online version in colour.)

Two 5 MP digital cameras were set up and calibrated for three-dimensional DIC on one side of the panel, which now had a finer speckle pattern applied owing to the higher resolution of the recording apparatus and the smaller size of the samples, as per standard image correlation practices [33]. In all cases, the imaged face was the skin, which was originally facing the explosive charge. A schematic of the experimental test set-up is shown in figure 14.

The panels were compressed at a constant displacement rate of 2 mm min^−1^. The panels were compressed until either full delamination occurred sharply, or the load dropped to below 20% of the maximum. Images of the panels were taken at a rate of 1 Hz, measuring the displacement of the front face of the panel, but not recording dynamic unloading of the panel due to fast fracture. The blast panels were tested in edgewise compression under the assumption that the naval vessel will only be subjected to one blast event, meaning that integrity of the hull need only be maintained until such time as repairs can take place. Owing to extensive core cracking during blast causing a discontinuity between the two facesheets, it is expected that flexural stiffness becomes minimal after blast, a prediction that can be verified with further testing. Edgewise compressive strength, on the other hand, should be much less affected by the blast (up to the buckling load) owing to the facesheets providing the majority of the structural input. For this reason, the test specimens were deemed to have ‘failed’ in the residual edgewise compression strength tests when buckling occurred. If these materials were structural members, then they may be expected to maintain a certain amount of static compressive load, and therefore this is of interest.

### (b) Results and analysis

**Figure 15.**
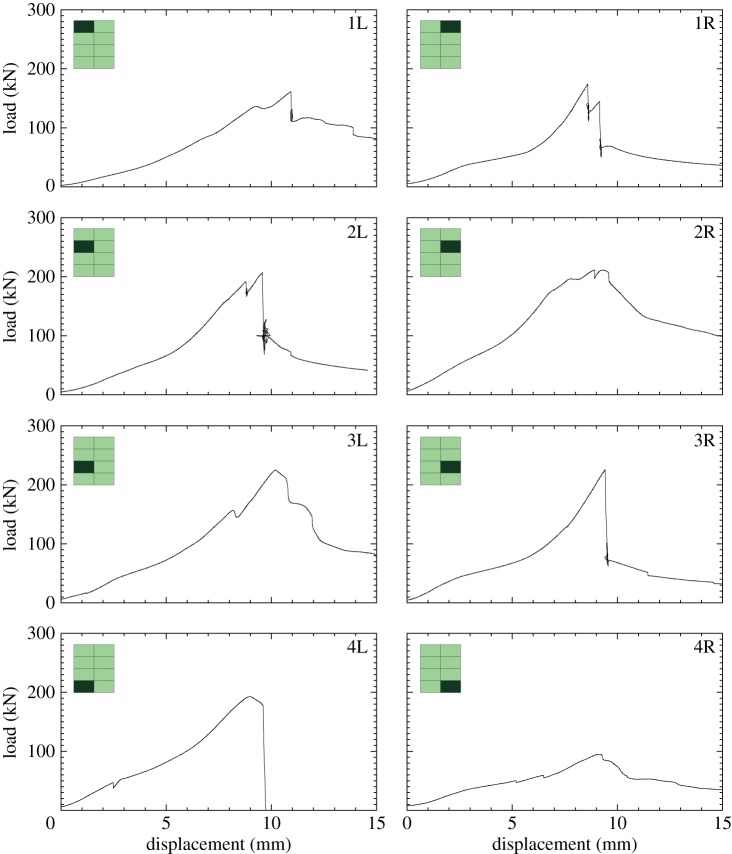
Force–time data for each sample in GL1. Data for each subpanel are shown in the position of where it lay in the global blast panel as viewed from rear of panel. The inset shows the location of the subpanel within the GFRP panel. (Online version in colour.)

**Figure 16.**
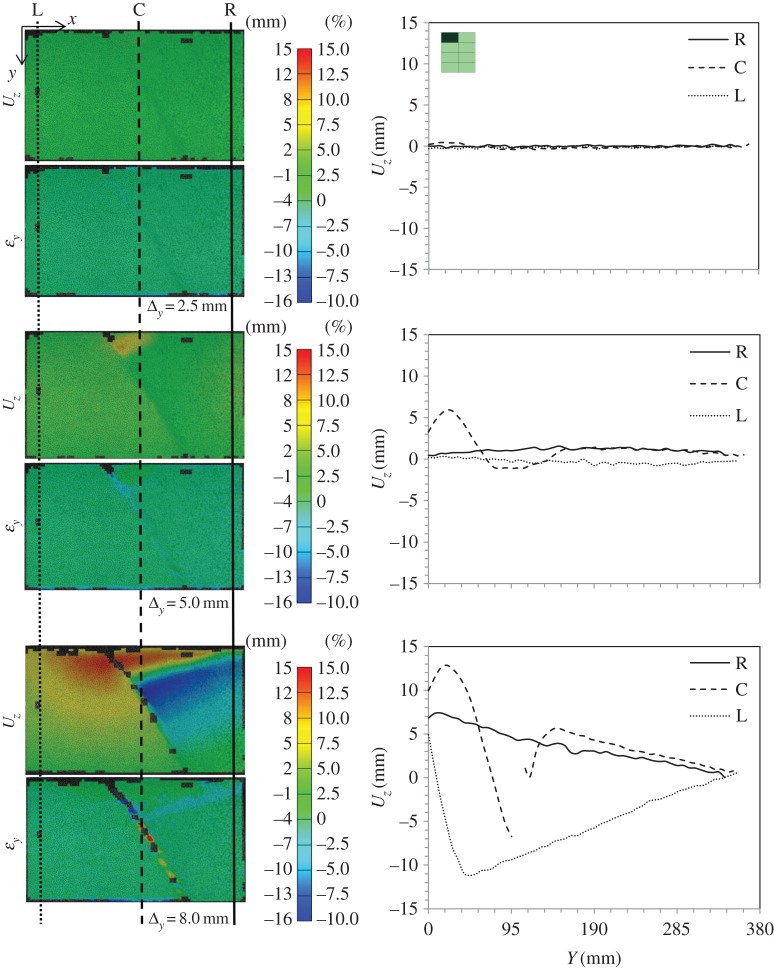
DIC analysis for subpanel 1L (top left when viewed from rear) in global blast panel GL1: out-of-plane displacement, *U*_*z*_, and strain in the *y*-direction, *ε*_*y*_; and section plots showing the variation of *U*_*z*_ along the height of the panel at three locations along the length—right (R), centre (C) and left (L). Note the subpanel dimensions were 635×400 mm; however, a window height of 360 mm was imaged. (Online version in colour.)

**Figure 17.**
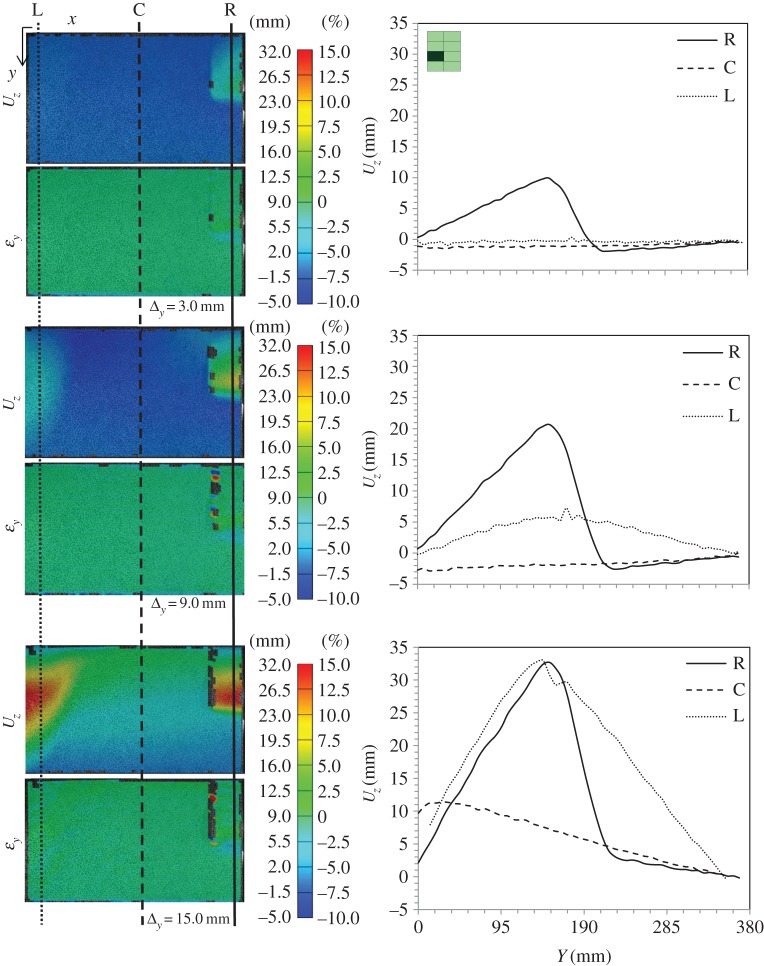
DIC analysis for subpanel 3L in global blast panel GL1: out-of-plane displacement, *U*_*z*_, and strain in the *y*-direction, *ε*_*y*_; and section plots showing the variation of *U*_*z*_ along the height of the panel at three locations along the length—right (R), centre (C) and left (L). (Online version in colour.)

**Figure 18.**
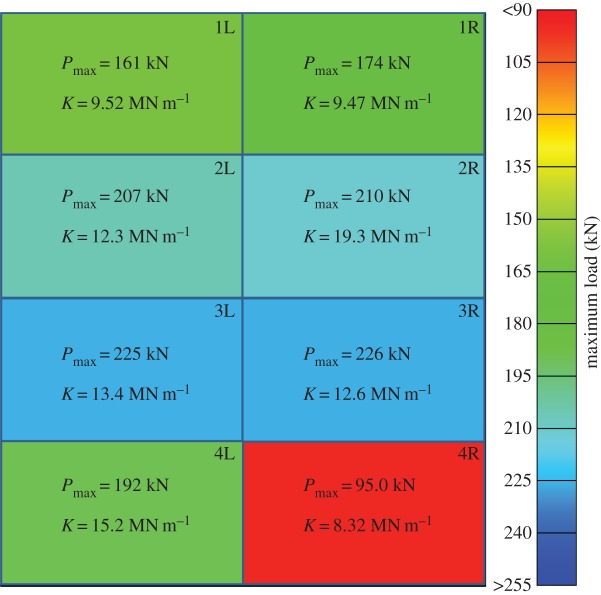
Graphic showing, as a greyscale (colour) plot, how ultimate load-bearing capacity varied with location of subpanel on the global GFRP blast panel as viewed from rear. (Online version in colour.)

Figure 15 shows a summary of all the load–displacement plots taken for each subpanel of GL1 tested, with each location of the subpanel within the global blasted panel labelled. It was observed in the glass specimens that the ultimate compressive load was greater for those panels which were situated in positions 2 and 3. The damage here was observed to be less severe than the bottom of the panels. The damage in the centre panels was also noted to be predominantly at the top and bottom, and the DIC data from the blast test (figures 16 and 17) suggested that core cracks travelled vertically, in line with the direction of the compressive load. It can be seen in figure 18 that higher ultimate loads were withstood by the panels in positions 2 and 3, and that, particularly in position 4R, a much lower load was attained before ultimate failure. These panels demonstrated cracks travelling across the loading direction, and 4R showed the most damage of any of the glass-fibre panels.

Figure 16 shows DIC images, which have been extracted to illustrate out-of-plane displacement, *U*_*z*_, of the front GFRP skin, as well as strain in the vertical direction, *ε*_*y*_, on the front face. The idea here is that, during the early stages of the loading of the subpanels, the DIC contours can tell the user what kind of subsurface damage is present. A simple concept is proposed such that, if there is a localized strain field but a uniform displacement trace, then subsurface cracks in the core are causing this strain concentration. If there is a bulge in the *U*_*z*_ plot, relative to the global behaviour, and a strain concentration, then there is likely to be a debond present (in addition to any cracks or otherwise). Therefore, this analysis has been used to clarify what damage was present, reaffirming edge inspections or correcting them. Graphs of out-of-plane displacement along the height of the subpanels are shown for the edges and centre of these subpanels to illustrate the bulging effect or uniformity where present. Plots are shown for 1L in figure 16 and 3L in figure 17 for various compressive loading stages labelled with reference to the full load–displacement plots given in figure 14.

Clearly visible from these images are residual cracks that were in place in the front glass sheet from blast testing. Also visible in these DIC images is the fact that wrinkling has taken place in the front skin, mainly due to discontinuity across the facesheet as a result of the skin cracking. This is an interesting feature, as it shows that the two facesheets still maintain some connection through the foam, so not all out-of-plane displacement is away from the centreline.

**Figure 19.**
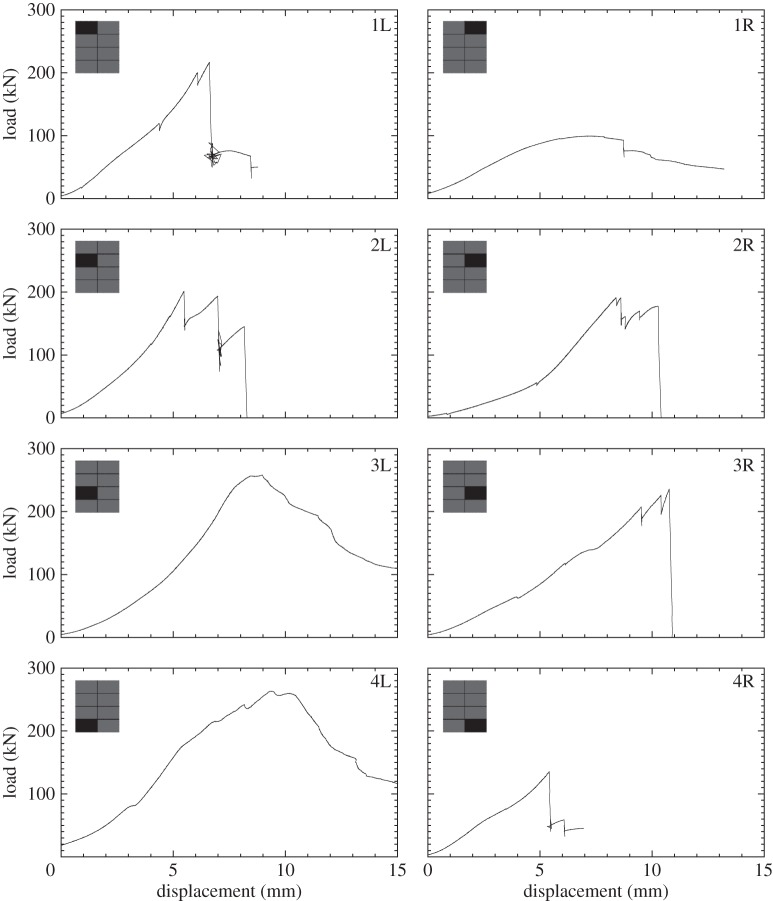
Force–time data for each sample in CA1. Data for each subpanel are shown in the position of where it lay in the global blast panel as viewed from rear. The inset shows the location of the subpanel within the CFRP panel.

**Figure 20.**
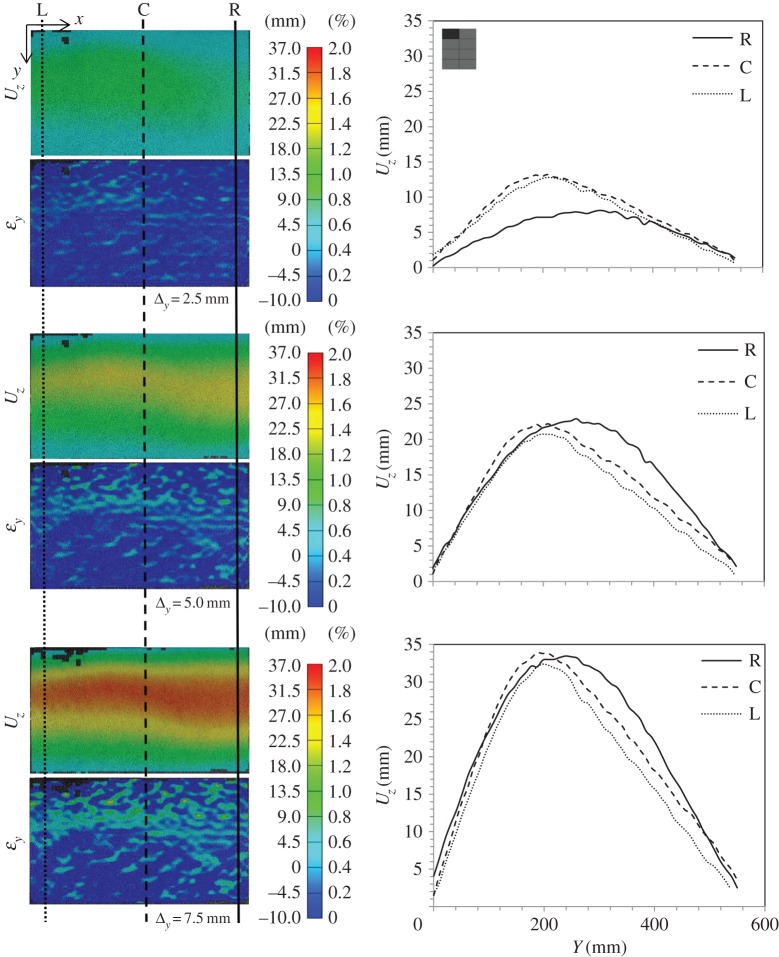
DIC analysis for subpanel 1L in global blast panel CA1: out-of-plane displacement, *U*_*z*_, and strain in the *y*-direction, *ε*_*y*_; and section plots showing the variation of *U*_*z*_ along the height of the panel at three locations along the length—right (R), centre (C) and left (L). (Online version in colour.)

**Figure 21.**
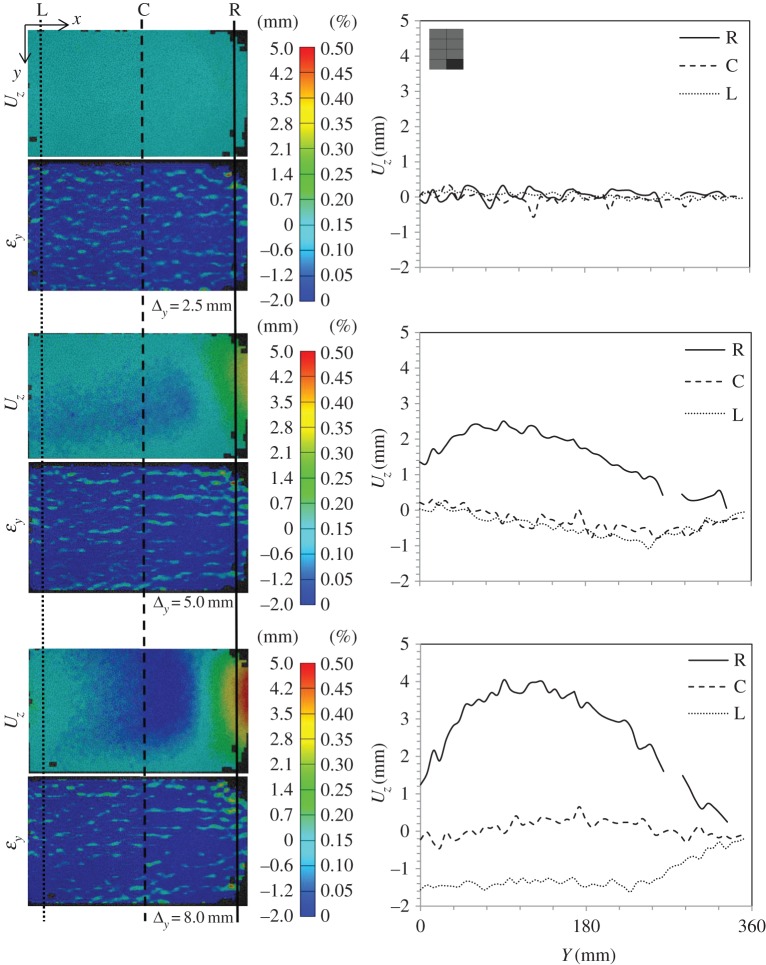
DIC analysis for subpanel 4R in global blast panel CA1: out-of-plane displacement, *U*_*z*_, and strain in the *y*-direction, *ε*_*y*_; and section plots showing the variation of *U*_*z*_ along the height of the panel at three locations along the length—right (R), centre (C) and left (L). (Online version in colour.)

Figure 19 shows a summary of the load–displacement plots for the subpanels of CA1 tested. Figures 20 and 21 show the DIC for out-of-plane displacement in the CA1 subpanels 1R and 4R, respectively. The point during each test where the plots have been extracted are marked in terms of compressive displacement relating to the plots in figure 19. Similar to those figures produced for the GFRP subpanels, each compressive stage shows DIC images to illustrate the variation of out-of-plane motion and *ε*_*y*_ along the height of the panel front. The carbon skins suffered minimal damage during blast testing, which can be seen from the even displacements in the DIC images. However, core cracking and debonding were present in these test samples. In CA1-4R, this can be seen by the high out-of-plane deflection and relatively uniform strain plots on the right-hand edge, which corresponds to the core cracking damage.

**Figure 22.**
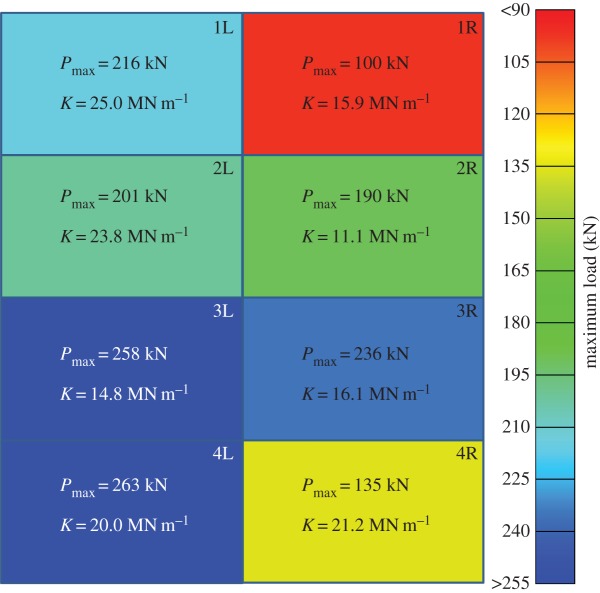
Graphic showing, as a greyscale (colour) plot, how ultimate load-bearing capacity varied with location of subpanel on the global GFRP blast panel as viewed from rear. (Online version in colour.)

Ultimate load-bearing capacity data are presented for the CFRP panels in figure 22. It can be seen again that the residual strength of the panels corresponds to the damage severity and orientation observed from the edges of the panels and interpreted from the DIC blast data. In this case, it can be noted that panel 4L demonstrates a very high ultimate compressive strength, and that the corresponding corner, 1R, demonstrates a very low compressive strength. This corresponds to the amount of visible damage to the panels, and is indicative of the unequal loading shown in the DIC blast data due to the constraint of the panel. Otherwise, a reasonably high compressive strength was still observed at the centre of the carbon-fibre panel, and locations of lower strength were observed at the upper and lower positions.

## 5. Discussion

It was shown that during the blast the CFRP-skinned sandwich panels provided a greater resistance to the blast-wave impact, deflecting to a lower 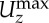 compared with the GFRP-skinned target. The post-blast damage inspection showed CA1 and CA2 to have suffered minimal to no visible skin damage, but comparable severity of core damage to GL1 and GL2. The GFRP targets both suffered significant front-face skin cracks. The cracks were seen to originate in the corners of the targets where there is a high degree of restraint on the sample. It is here that the shear waves superimpose after rebound from the boundaries to initiate core failures, leading to compressive skin failures once sufficiently detached from the core to enable the failure strain, approximately 1.5%, to be reached.

The amount of core damage was approximately the same across the panel, but the skin damage was localized. This can be expected on a structure owing to the uneven loading that can develop during a blast. It is expected that the central portion of the test fixture towards the bottom would see the greatest initial loading. This is evident from the *U*_*z*_ plots during each blast for the targets in figures 6 and 8. However, composite structures exhibit compliance; therefore the pressures do not build up necessarily to their theoretical maxima based on geometry alone. GL1 was seen to suffer the most damage at the bottom corner closest to the centre of the fixture (4R), whereas CA1 was seen to suffer most damage in the opposite corner (1R). When the blast wave hits the targets, there are a series of transmitted waves and reflected waves. The transmitted waves are either through-thickness surface waves or a combination of shear waves. Upon impact, the bottom-most central corner (GL1-4R and CA1-4L) will be loaded first and see the initial peak loading relative to the whole target, which is also being loaded by the blast. There is a period of build-up during the loading phase, particularly on a non-infinitely rigid surface. The surface waves travelling along the panel are transmitted along the lengths towards other regions of the target, i.e. opposite corners. The faster wave speed in the CFRP targets allows the waves to propagate to the other boundaries quicker than in the GFRP targets. Therefore, damage builds up greatly in the CFRP targets away from the centre. However, in the GFRP targets, the magnitude of the blast wave is sufficient to breach the failure strain of the fibres (especially once support from the core is lost due to shear failures), and so energy dissipation to remote areas from peak-loaded ones is minimized. Energy is lost due to other failure mechanisms in the GFRP sandwich panels such as gross skin failure, rather than simply core damage. Therefore, the more significantly damaged area is focused in the GFRP targets to where the most force is expected to be applied and not necessarily focused in the CFRP targets. This concept is shown in figures 18 and 22.

The GFRP targets appeared to sustain a broad degree of damage, as did the CFRP targets. For a blast-resistant structure, it is advantageous to understand the residual strength of the structure post-blast, as well as what level of blast it can sustain prior to catastrophic failure, e.g. rear-skin perforation. The edgewise compression tests represent one of a few types of loading conditions that would be of interest to study from the point of view of the residual strength in structures; others include flexure and normal impact or compressive loading.

**Figure 23.**
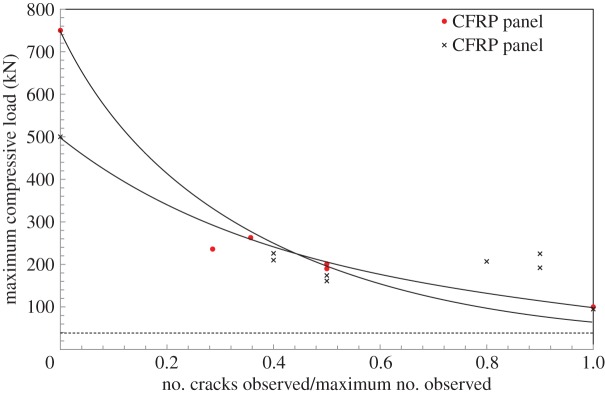
Plot showing compressive load against normalized number of cracks recorded prior to compression testing. Dashed line represents the theoretical minimum compressive load to crush the core and buckle the skins. (Online version in colour.)

A number of trends and hypotheses can be drawn from the raw data. However, the importance of core cracking, particularly those normal to the loading direction, can lead to significant reduction in stiffness and load-bearing capacity. The graph shown in figure 23 plots the normalized number of cracks, i.e. the total number of cracks observed visually from each edge of the subpanel divided by the greatest number observed, all plotted against the peak compressive load. The peak values of compressive load were obtained from testing non-blasted materials.

Referring to figure 23, it is seen that the CFRP targets have a much higher initial maximum load-bearing capacity (which is taken to be the buckling load) than the GFRP targets. However, based on the stiffer fibres in CFRP and the improved blast resistance, resulting in greater degree of blast energy absorption, the relative reduction in load-bearing capacity was greater for the CFRP targets compared with the GFRP targets. Furthermore, a stiffer material experiences a higher load (or resistance to impact); therefore the apparent damage induced in the core and interfaces is understandable compared with a more compliant material such as GFRP.

Both targets seem to reach a similar lower level of strength once some small amount of damage is incurred. The graph, however, goes beyond the *x*-axis plotted, i.e. the panels can damage further than shown in this paper, to a theoretical maximum damage where the panels are loaded purely to compress the foam core and buckle the skins. This minimum compressive load is shown as the dotted asymptote at the bottom of the graph in figure 23.

## 6. Conclusion

CFRP sandwich panels were shown to have a greater blast resistance, in terms of their deflection under severe blast to a lower peak out-of-plane displacement, than the GFRP equivalent targets. Damage and failure mechanisms were identified to be shear failure initiation in the core and its inherent propagation through the core and core–skin interface. Skin failures were clearly observed in the GFRP targets, forming around areas of complete skin-to-skin core shear failure, with some skin cracking seen to occur to a lesser degree in the CFRP targets.

The residual strength of the two constructions was evaluated under compression loading. The percentage drop in the CFRP targets was greater than in the GFRP targets. It was shown that, once a certain level of damage is inflicted by the 100 kg TNT blast, there is a drop in load-bearing capacity. Carbon fibres are much stronger than glass fibres, but both remain susceptible to impact damage, and so the relative drop in strength was seen to be greater in the CFRP sandwich panels. The trends observed here indicate that, if residual strength is a key design factor after severe blast, then a drop of two-thirds of the residual compressive strength in CFRP and half in GFRP needs to be taken into account in design.
